# Evaluation of the effectiveness of total hip arthroplasty with preservation of the piriformis muscle using the posterolateral approach: A retrospective multicenter study

**DOI:** 10.1097/MD.0000000000048771

**Published:** 2026-05-15

**Authors:** Kaiyuan Zheng, Haiqi Ding, Xiaoyong Wang, Changzhi Huang, Bei Li, Naijian Dong, Jie Xu, Jiuzao Lin, Jiagu Huang

**Affiliations:** a Department of Joint and Sports Medicine, Ningde Clinical Medical College of Fujian Medical University, Ningde, Fujian, China; b Department of Joint and Sports Medicine, Ningde Municipal Hospital, Ningde, Fujian, China; c Department of Orthopedics, Fuzhou University Affiliated Provincial Hospital, Shengli Clinical Medical College of Fujian Medical University, Fuzhou, Fujian, China; d Department of Orthopedics, Fujian Provincial Hospital, Fuzhou, Fujian, China; e Department of Orthopedics, Fujian Provincial Clinical Medical Research Center for Spinal Nerve and Joint Diseases, Fuzhou, Fujian, China.

**Keywords:** piriformis, posterolateral approach, total hip arthroplasty

## Abstract

To investigate the influence of preserving the piriformis muscle during surgery on postoperative hip joint function recovery and the incidence of dislocation following total hip arthroplasty (THA). This retrospective multicenter study collected clinical data and follow-up information from patients with hip osteoarthritis or femoral head necrosis who underwent primary unilateral total hip arthroplasty at Ningde Municipal Hospital and Fujian Provincial Hospital between 2019 and 2024. A minimum follow-up of 12 months was required for inclusion. Patients were followed at 1 week, 1 month, 3 months, and 12 months after surgery. The choice of surgical approach was made by the attending surgeon according to preoperative planning, surgeon familiarity with the technique, and intraoperative exposure feasibility; it was not assigned by a study protocol. Patients were categorized into the posterolateral piriformis-sparing approach (Mis-PLA) group and the traditional posterolateral approach (PLA) group. A total of 420 patients were finally included, among whom 208 were in the Mis-PLA group and 212 were in the PLA group. All patients completed at least 12 months of follow-up. The preoperative Harris Hip Score was comparable between groups (PLA: 42.7 ± 7.9 vs Mis-PLA: 41.3 ± 8.2; *P* = .075). At 12 months, the Harris Hip Score remained similar between groups (PLA: 94.98 ± 2.12 vs Mis-PLA: 95.26 ± 1.85; *P* = .150). In repeated-measures analysis adjusted for age, sex, and body mass index, the group-by-time interaction was significant (Wald χ^2^ = 66.39, *P* < .001), and the Mis-PLA group remained associated with higher Harris Hip Scores at 1 week (β = 5.38, 95% CI: 3.61–7.15; *P* < .001) and 1 month (β = 3.22, 95% CI: 1.26–5.18; *P* = .001). In multivariable linear regression, Mis-PLA was independently associated with a higher 1-month Harris Hip Score (β = 2.12, 95% CI: 0.85–3.40; *P* = .001). The incidence of early postoperative hip dislocation was significantly lower in the Mis-PLA group than in the PLA group (0% vs 2.8%, *P* = .042). In this retrospective multicenter cohort, preservation of the piriformis muscle during the posterolateral approach was associated with reduced early dislocation and improved early functional recovery, without compromising 12-month functional outcomes. Preservation of the piriformis muscle during the posterolateral approach may be considered a feasible option to reduce early dislocation without compromising functional recovery.

## 1. Introduction

Hip replacement surgery is a pivotal technological advancement in modern orthopedics, extensively utilized for treating severe hip joint conditions like avascular necrosis of the femoral head, hip osteoarthritis, and femoral neck fractures. These conditions not only detrimentally impact patients’ quality of life but can also result in enduring pain and functional limitations. By replacing the deteriorated hip joint surface, hip replacement surgery reinstates joint function, alleviates pain, and enhances patients’ quality of life. The selection of a surgical approach stands out as a critical determinant of surgical success. Common approaches encompass the direct anterior approach, the Orthopadische Chirurgie Munchen approach, and the posterolateral approach (PLA). The posterolateral approach is widely favored for its clear surgical field and procedural convenience. Nevertheless, the handling of surrounding tissues, such as the piriformis muscle, within this approach significantly influences surgical outcomes and postoperative recovery.

The posterolateral approach is a widely utilized technique in hip replacement surgery that has undergone iterative refinement. Initially, in early PLA procedures, surgeons commonly excised external rotator muscle groups, such as the piriformis muscle, to enhance the surgical field and facilitate the operation. However, while this approach yielded a clear surgical field, it often led to complications like hip abductor muscle weakness and gait abnormalities postoperatively, thereby elevating the risk of dislocation.^[1]^ Presently, a substantial body of clinical evidence supports the notion that repairing the joint capsule and preserving the short external rotator muscle groups can enhance postoperative hip joint stability.^[2]^ We gradually recognized the significance of conserving the piriformis muscle and started investigating surgical techniques for its preservation. Our observations indicated that utilizing a posterolateral approach while preserving the piriformis muscle may decrease muscular injury.

Several studies have demonstrated the benefits of the posterolateral approach with piriformis muscle preservation on early postoperative recovery of hip joint function.^[3]^ Patients undergoing this procedure experience reduced postoperative pain, earlier ambulation initiation, and improved satisfaction with their recovery. For instance, Siddappa et al^[4]^ compared the outcomes of the posterolateral approach with and without piriformis muscle preservation and reported a significant decrease in the risk of hip dislocation when preserving the piriformis muscle compared with the conventional method of muscle transection. Another investigation evaluated the therapeutic impact of piriformis muscle preservation, revealing notable enhancements in postoperative Harris scores, visual analogue scale (VAS) scores, and joint function recovery.^[5]^ Nevertheless, preserving the piriformis muscle during surgery has been acknowledged as technically challenging because it limits exposure of the operative field and requires a high level of operator skill.^[6]^ Moreover, while this approach shows advantages in early postoperative function recovery, some studies suggest that there is minimal disparity in long-term efficacy between the 2 surgical methods.^[7]^

Based on these clinical observations, the present study further examined the efficacy of posterolateral total hip arthroplasty (THA) with preservation of the piriformis muscle. Through a comparative analysis of preserving versus cutting the piriformis muscle during surgery, this study assessed their respective impacts on postoperative hip function recovery, pain management, length of hospitalization, and related perioperative factors, aiming to furnish additional evidence for clinical application.

## 2. Methods

### 2.1. Patient selection

This study was designed as a retrospective multicenter cohort study. Clinical records of patients who underwent THA at Ningde Municipal Hospital and Fujian Provincial Hospital between September 2019 and September 2024 were reviewed. Eligible patients had hip joint disorders caused by osteoarthritis or femoral head necrosis that required primary unilateral THA and had complete follow-up for at least 12 months. Patients with severe systemic illness or contraindications to surgery were excluded. Exclusion criteria also included severe osteoporosis, previous hip revision, severe autoimmune-related hip disease such as rheumatoid arthritis or ankylosing spondylitis, proximal femoral deformity, severe acetabular dysplasia (Crowe III or IV), hepatorenal insufficiency, major cardiovascular or cerebrovascular disease, coagulation disorders, acute or chronic infectious disease, malignancy, neurological disorders, and a history of mental illness.

The study protocol was approved by the Ethics Committee of Ningde Municipal Hospital and the Ethics Committee of Fujian Provincial Hospital, respectively. Because this was a retrospective analysis of routine clinical practice, the surgical approach was not assigned by protocol; instead, the attending surgeon selected posterolateral piriformis-sparing approach (Mis-PLA) or PLA according to preoperative planning, surgeon familiarity with the technique, and intraoperative exposure feasibility. To reduce surgeon-related heterogeneity, 2 senior arthroplasty surgeons were involved in total, one at each participating center, and all procedures at each center were performed by the same surgeon, who had received standardized training in the Mis-PLA technique before study initiation.

### 2.2. Surgical methods

**Figure 1.**
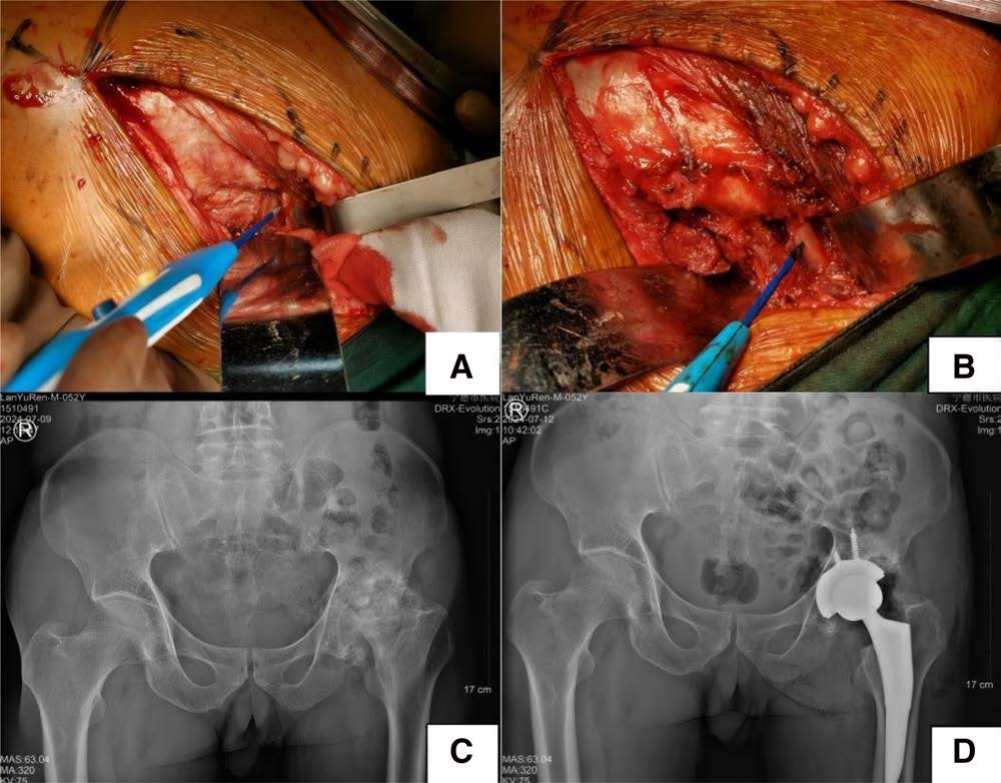
Male patient, 52 yr old, left DDH with OA, underwent total artificial replacement without cutting the piriformis muscle through the posterolateral approach. (A) The complete piriformis muscle was exposed during the operation. (B) The piriformis muscle was not severed during the operation. (C) Preoperative anteroposterior pelvic X-ray; (D) Postoperative anteroposterior X-ray of the pelvis. DDH = developmental dysplasia of the hip, OA = osteoarthritis.

Two senior arthroplasty surgeons were involved in total, one at each participating center, and all procedures at each center were performed by that surgeon who had received standardized training in the Mis-PLA technique prior to the study. This ensured that surgical steps, such as the exposure and preservation of the piriformis muscle, were standardized as much as possible across centers. Mis-PLA group: A posterolateral incision was made to access the hip joint. Sequentially, the skin, subcutaneous tissue, and fascia lata were incised. The gluteus maximus was bluntly separated following the muscle fibers’ direction. The greater trochanteric bursa was incised to expose the external rotator muscles, and the piriformis muscle was dissected bluntly. At the lower edge of the piriformis muscle, the conjoined tendon of the short external rotators (superior gemellus, inferior gemellus, and part of the quadratus femoris) was transected at its insertion on the greater trochanter of the femur, while protecting the sciatic nerve. Subsequently, the hip joint was dislocated, and the femoral neck was transected 8 to 10 mm above the lesser trochanter. The femoral head was removed and the acetabulum was prepared according to standard procedures. Trial prostheses were then used to assess the suitability and stability of the implant. After confirmation of satisfactory trial reduction and stability, the definitive prosthesis was implanted into the acetabulum. The medullary cavity was opened along the femoral neck’s long axis, reamed sequentially to determine the appropriate size for the femoral prosthesis. A suitable biological prosthesis was selected, tapped to the correct depth, and a trial head of suitable size was inserted. Subsequent flexion and extension movements confirmed good stability, mobility of the hip joint, and moderate soft-tissue tension. After thorough irrigation with iodine and normal saline, the hip joint was dislocated again, and a matching ball head was installed. The muscle groups and joint capsule were repositioned, and each layer was sutured sequentially. The counts of gauze and instruments were verified before complete layer-by-layer wound closure and application of a sterile dressing. A representative case is shown in Figure 1.

PLA group: All external rotator muscle groups, including the piriformis muscle, were cut off, and the remaining surgical steps remained the same as those in the Mis-PLA group.

### 2.3. Observation indicators

Primary outcome measures: Hip joint function was assessed using the Harris Hip Score preoperatively, as well as at 1 week, 1 month, 3 months, and 1 year postoperatively. Patients were monitored throughout the follow-up period for the occurrence of postoperative dislocation, with particular attention to early dislocation within 3 months. Secondary outcome measures include assessing the length of the surgical incision, as well as the duration of the operation (defined as the interval from skin incision to completion of prosthesis installation, measured in minutes). Additionally, document VAS scores on postoperative days 1, 2, and 3. Quantify intraoperative blood loss and document any postoperative complications. Evaluate acetabular anteversion angle, acetabular abduction angle, and combined anteversion angle in both study groups.

### 2.4. Statistical methods

Statistical analyses were performed using SPSS software (version 23.0). Continuous variables are presented as mean ± standard deviation, and categorical variables are presented as counts and percentages. Baseline and perioperative between-group comparisons were performed using independent-sample t tests for continuous variables and the chi-square test or Fisher exact test for categorical variables, as appropriate. Fisher exact test was used for outcomes with small cell counts, including early hip dislocation. Because this was a retrospective exploratory study, no a priori sample-size or power calculation was performed; the study size was determined by the number of eligible cases available during the study period. For longitudinal analysis of Harris Hip Score, a generalized estimating equation model with Gaussian distribution and exchangeable working correlation structure was fitted, including group, time, and the group-by-time interaction, with adjustment for age, sex, body mass index (BMI), and study center. In addition, between-group comparisons at each prespecified follow-up time point (1 week, 1 month, 3 months, and 12 months) are presented as descriptive supplementary comparisons. To further evaluate early functional recovery, a multivariable linear regression model was fitted for the 1-month Harris Hip Score with adjustment for surgical group, age, sex, BMI, study center, and preoperative Harris Hip Score. Two-sided *P* < .05 was considered statistically significant.

## 3. Results

### 3.1. Cohort characteristics and baseline comparability

**Figure 2.**
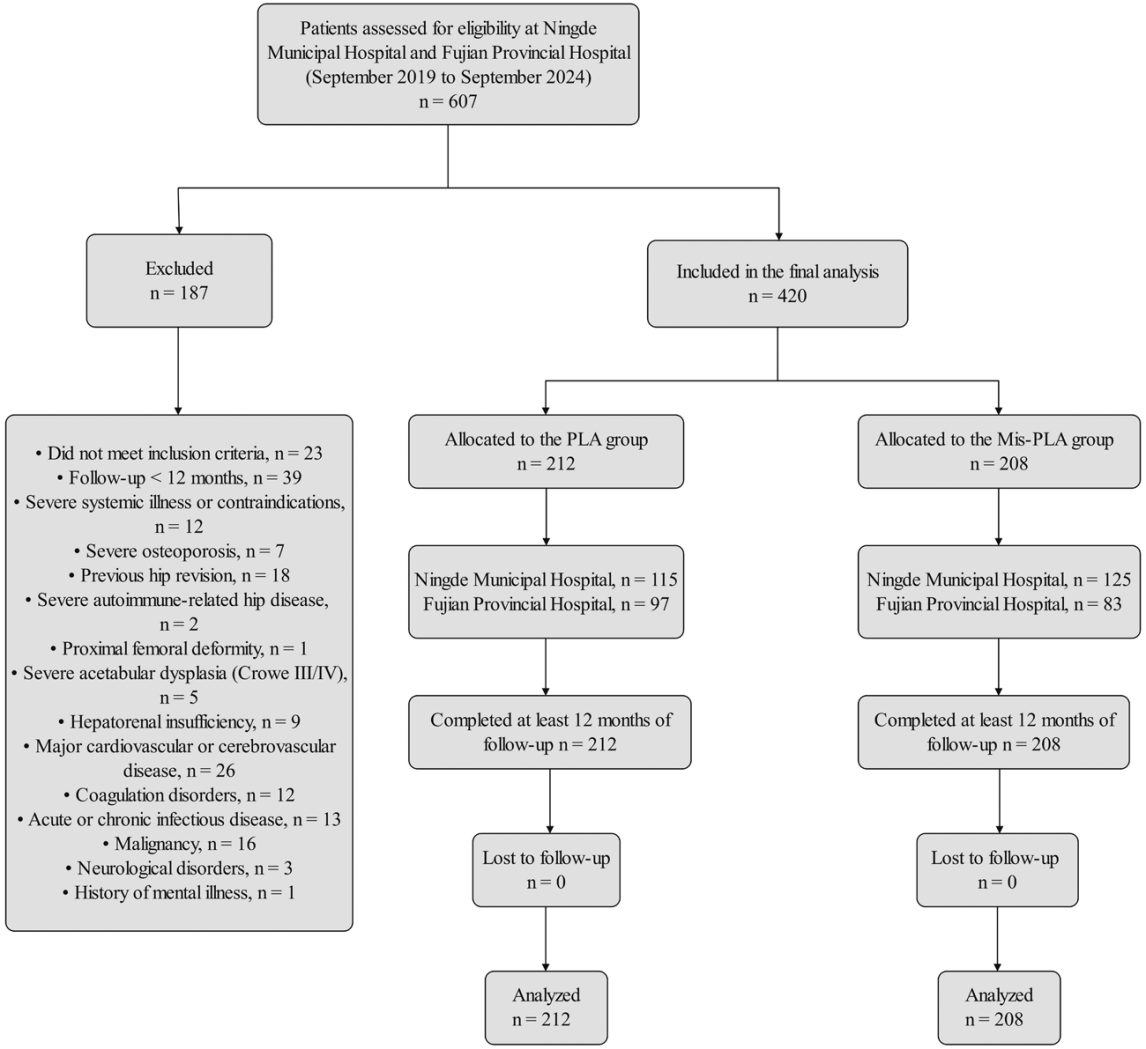
STROBE-style flow diagram of patient enrollment and group allocation. STROBE = Strengthening the Reporting of Observational Studies in Epidemiology.

A total of 420 patients met the study criteria and were included in the final analysis, including 208 patients in the Mis-PLA group and 212 patients in the PLA group. Among the 420 included patients, 240 were treated at Ningde Municipal Hospital (115 in the PLA group and 125 in the Mis-PLA group), and 180 were treated at Fujian Provincial Hospital (97 in the PLA group and 83 in the Mis-PLA group). The distribution of study center did not differ significantly between the 2 groups (χ^2^ = 1.468, *P* = .226). All included patients completed follow-up to at least 12 months. There were no statistically significant differences in mean age, sex distribution, BMI, or preoperative Harris score between the 2 groups, indicating comparable baseline characteristics. Likewise, no statistically significant differences were observed in incision length, operation time, or intraoperative blood loss (Table 1). Strengthening the Reporting of Observational Studies in Epidemiology-style flow diagram of patient enrollment and group allocation is shown in Figure 2.

**Table 1:** General data of patients in the 2 groups [$\bar{x}\pm s$, n(%)].

Indicators	PLA (n = 212)	Mis-PLA (n = 208)	*t*/χ^2^ value	*P* value
Age (yr)	72.10 ± 3.35	71.80 ± 4.01	0.832	.406
Gender (Male/Female)	109/103	102/106	0.237	.626
BMI (kg/m^2^)	24.5 ± 2.4	24.3 ± 2.5	0.836	.403
Preoperative Harris score	42.7 ± 7.9	41.3 ± 8.2	1.782	.075
Surgical time (min)	45.4 ± 5.9	46.4 ± 5.6	1.781	.076
Incision length (cm)	9.5 ± 1.0	9.4 ± 1.3	0.885	.377
Intraoperative blood loss (mL)	250.8 ± 50.2	241.8 ± 45.1	1.932	.054
Ningde MunicipalHospital/Fujian Provincial Hospital	115/97	125/83	1.468	.226

BMI = body mass index, Mis-PLA = posterolateral piriformis- sparing approach, PLA = posterolateral approach.

### 3.2. Intraoperative radiographic parameters

Comparison of acetabular anteversion angle, acetabular abduction angle, and combined anteversion angle between the groups revealed no statistically significant differences in intraoperative measurements (Table 2).

**Table 2:** Comparison of intraoperative angles.

Angle	PLA (n = 212)	Mis-PLA (n = 208)	*t* value	*P* value
Acetabular anteversion angle	20.79 ± 4.96	19. 92 ± 4.58	1.866	.062
Acetabular abduction angle	44.46 ± 4.57	43.65 ± 5.20	1.697	.090
Combined anteversion angle	35.82 ± 5.27	35.46 ± 4.64	0.743	.458

Mis-PLA = posterolateral piriformis- sparing approach, PLA = posterolateral approach.

### 3.3. Postoperative pain scores

There were no statistically significant differences in VAS scores between the 2 groups on postoperative days 1, 2, and 3 (Table 3).

**Table 3:** Postoperative pain scores of the affected hip joint ($\bar{x}\pm s$).

VAS score	PLA (n = 212)	Mis-PLA (n = 208)	*t* value	*P* value
Postoperative day 1	5.0 ± 1.4	4.9 ± 0.9	0.868	.385
Postoperative day 2	3.8 ± 0.7	3.7 ± 0.8	1.364	.173
Postoperative day 3	3.1 ± 0.6	3.0 ± 0.7	1.572	.117

Mis-PLA = posterolateral piriformis- sparing approach, PLA = posterolateral approach, VAS = visual analogue scale.

### 3.4. Functional evaluation

In the descriptive between-group comparisons at prespecified follow-up time points, the Mis-PLA group had significantly higher Harris Hip Scores at 1 week and 1 month after surgery than the PLA group (both *P* < .001), whereas no significant between-group differences were observed at 3 months or 12 months (Table 4). The generalized estimating equation model showed a significant group-by-time interaction (Wald χ^2^ = 66.39, *P* < .001), indicating that postoperative functional recovery patterns differed between the 2 groups over time. After adjustment for age, sex, BMI, and study center, the Mis-PLA group remained associated with higher Harris Hip Scores at 1 week (β = 5.38, 95% CI: 3.61–7.15; *P* < .001) and 1 month (β = 3.22, 95% CI: 1.26–5.18; *P* = .001). In multivariable linear regression, Mis-PLA also remained independently associated with a higher 1-month Harris Hip Score after additional adjustment for preoperative Harris Hip Score (β = 2.12, 95% CI: 0.85–3.40; *P* = .001). (Table 5)

**Table 4:** Postoperative functional scores of the affected hip joint ($\bar{x}\pm s$).

Harris scores	PLA (n = 212)	Mis-PLA (n = 208)	*t* value	*P* value
1 wk	78.12 ± 4.76	81.28 ± 5.29	6.438	<.001
1 mo	85.28 ± 6.88	88.23 ± 6.23	4.603	<.001
3 mo	92.28 ± 7.18	93.45 ± 5.38	1.887	.059
12 mo	94.98 ± 2.12	95.26 ± 1.85	1.441	.150

Mis-PLA = posterolateral piriformis- sparing approach, PLA = posterolateral approach.

**Table 5:** Repeated-measures and multivariable analysis of Harris Hip Score.

Analysis	Comparison/ variable	Effect estimate	95% CI	*P* value
Repeated-measures GEE	Overall group-by-time interaction	Wald χ^2^ = 66.39	–	<.001
Repeated-measures GEE	Mis-PLA at 1 wk vs PLA	β = 5.38	3.61–7.15	<.001
Repeated-measures GEE	Mis-PLA at 1 mo vs PLA	β = 3.22	1.26–5.18	.001
Multivariable linear regression	Mis-PLA for 1-mo Harris score	β = 2.12	0.85–3.40	.001

CI = confidence interval, GEE = generalized estimating equations, Mis-PLA = posterolateral piriformis- sparing approach, PLA = posterolateral approach.

### 3.5. Postoperative complications

In the postoperative period, the PLA group exhibited 6 cases (2.8%) of early hip joint dislocation, 10 cases (4.7%) of deep venous thrombosis, and 0 cases (0%) of nerve injury. In contrast, the Mis-PLA group had 0 cases (0%) of early hip joint dislocation, 9 cases (4.3%) of deep venous thrombosis, and 0 cases (0%) of nerve injury. Analysis revealed no statistically significant difference in overall complications between the 2 groups (*P* > .05), as detailed in Table 6.

**Table 6:** Comparison of postoperative complications [n(%)].

Complications	PLA (n = 212)	Mis-PLA (n = 208)
Early hip dislocation	6	0
Deep venous thrombosis	10	9
Nerve injury	0	0
Total	16 (7.5%)	9 (4.3%)
*P* value	.163	

Mis-PLA = posterolateral piriformis- sparing approach, PLA = posterolateral approach.

**Figure 3.**
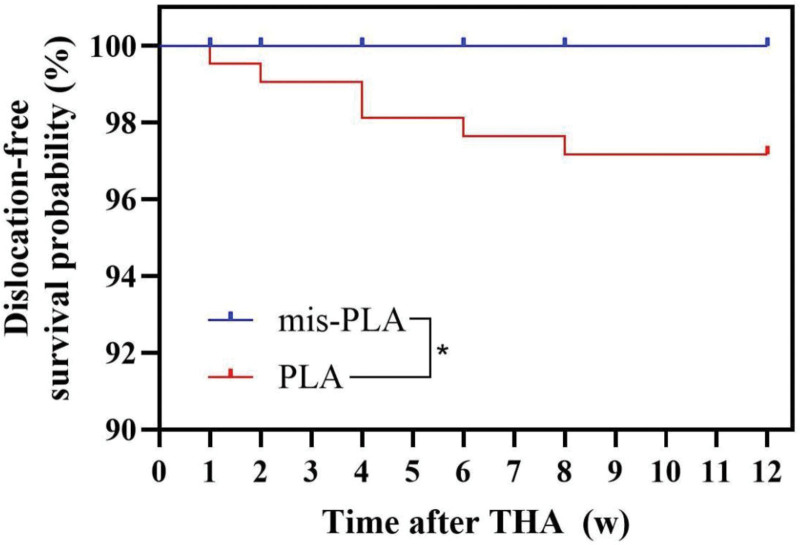
Kaplan–Meier curves for early postoperative dislocation-free survival comparing the Mis-PLA and PLA groups. Mis-PLA = posterolateral piriformis- sparing approach, PLA = posterolateral approach.

Specifically, the incidence of early hip joint dislocation was significantly higher in the PLA group (6 cases, 2.8%) compared to the Mis-PLA group (0 cases, 0%), with a statistically significant difference (*P* = .042). Kaplan–Meier curves for early postoperative dislocation-free survival comparing the Mis-PLA and PLA groups are shown in Figure 3.

## 4. Discussion

The posterolateral approach is a commonly employed technique in THA due to its simplicity, reduced blood loss, and shorter duration. However, it necessitates the division of key external rotator muscles, such as the piriformis muscle, and the posterior joint capsule, resulting in diminished muscle tension and potential muscle atrophy. This surgical approach results in significant trauma and destabilizes the joint, increasing the risk of early dislocation and hindering postoperative hip joint recovery.^[8]^ While Mis-PLA surgical procedures resemble those of PLA, the joint capsule is incised between the piriformis and superior gemellus muscles during the transection of external rotators. This approach preserves the piriformis muscle and minimizes soft tissue trauma. The piriformis muscle, the largest among the 6 short external rotators spanning from the sacrum to the greater trochanter of the hip joint, attaches to the upper edge of the greater trochanter slightly anterior to its tip, nestled between the thickened portions of the gluteus medius and gluteus minimus tendons. Functionally, the piriformis muscle envelops the posterior aspect of the femoral head, acting as a mechanical barrier against excessive axial internal rotation during weight-bearing to optimize hip joint loading and alignment. In a flexed position, it functions as an external rotator and abductor of the hip joint, contributing to the stabilization of the femoral head within the acetabulum.^[9]^

The study findings revealed a higher early dislocation rate of 2.8% in the PLA group compared to the Mis-PLA group following piriformis muscle preservation, with a statistically significant difference (*P* < .05). Specifically, all 6 dislocated cases in the PLA group were posterior dislocations, with 4 resulting from falls and 2 from excessive activities like internal rotation, flexion-extension, and adduction. This aligns with existing literature,^[10]^ indicating a higher dislocation rate post-PLA compared to other approaches. Notably, there were no significant differences in acetabular anteversion angle, abduction angle, and combined anteversion angle between the groups (*P* > .05), with all acetabular placement angles falling within the safe zone proposed by Lewinnek^[11]^ (abduction angle 30–50°, anteversion angle 5–25°), and combined anteversion angles controlled between 25 to 50°.^[12]^ Prosthetic angles and positions were accurately placed in both groups, with no clear association found between early postoperative dislocation in the PLA group and prosthetic angle. Preserving the piriformis muscle in THA with a posterolateral approach is suggested to reduce early dislocation incidence and enhance postoperative hip joint functional recovery. Maintaining the original hip joint anatomical structure during surgery to minimize soft tissue damage can aid in preserving soft tissue balance, enhancing mechanical blocking ability, and improving stability. However, PLA treatment may lead to increased trauma, potentially resulting in early dislocation and impacting hip joint functional recovery, consistent with findings by Moussallem et al.^[3]^ According to anatomical principles, preserving the piriformis muscle correlates with lower postoperative dislocation rates. Some studies^[13]^ have indicated that even if the piriformis muscle is incised and repaired in situ, it compromises the elasticity of posterior soft tissues, destabilizing the hip joint’s posterior soft tissues, and elevating the risk of posterior hip dislocation.

Rehabilitation and restoration of hip joint function post total hip arthroplasty (THA) are key research areas.^[14]^ Regarding functional recovery, the main advantage of the Mis-PLA technique was observed in the early postoperative period. Both the unadjusted comparisons and the adjusted repeated-measures/ multivariable analyses supported significantly higher Harris Hip Scores at 1 week and 1 month in the Mis-PLA group. Over time, as soft tissues healed, no significant functional differences were observed between the 2 groups at 3 months and at 12 months. By incorporating intraoperative reconstruction of the joint capsule and external rotator muscles on the greater trochanter, posterior stability was augmented, reinforcing the posterior mechanical blocking effect.^[15]^ In a study by Tarasevicius et al,^[16]^ outcomes from 276 THA cases were reported. Among these, 134 cases underwent soft tissue repair involving fixation of the piriformis muscle and joint capsule to the greater trochanter. The soft tissue repair group exhibited a lower incidence of joint dislocation (2.0%) compared to the non-repair group (5.0%). Currently, there is no relevant research clearly indicating whether preserving the piriformis muscle and repairing the joint capsule and external rotator muscles can relieve the post-operative position restrictions. Therefore, we still follow the standard posterior approach to limit the hip joint position after surgery.

No statistically significant differences were observed in operation time and intraoperative blood loss between the 2 groups. In the Mis-PLA group, preserving the piriformis muscle during surgery limited the surgical exposure. The intact piriformis muscle, with a certain level of tension, increased the difficulty in handling the femoral side compared to PLA. Nevertheless, the posterolateral approach demonstrated significant superiority in terms of hip joint exposure and operational complexity. In elderly patients, the piriformis muscle’s tension was relatively low, allowing for adequate field exposure without a notable disparity in operation time between the groups. However, patients with severe scar contracture of perijoint soft tissues or proximal femoral deformities posed challenges in field exposure. In such scenarios, decisive excision of the piriformis muscle during surgery was necessary to enhance visibility, prevent sciatic nerve injury, maintain proper prosthesis placement angles, and mitigate intraoperative and postoperative risks. A proximally curved femoral stem may be beneficial when preserving the piriformis muscle. When tension in the piriformis muscle is present, the arc design of the prosthesis’s proximal section can prevent damage to both the piriformis and gluteus medius muscles. Additionally, positioning the prosthesis as laterally as possible during the opening and reaming of the proximal femur can minimize varus displacement caused by soft tissue interference during implantation.

Numerous factors influence intraoperative blood loss, including operation duration, intraoperative blood pressure management, use of hemostatic medications perioperatively, and intraoperative soft tissue manipulation. Prior to surgery, neither patient group exhibited thrombocytopenia or coagulation abnormalities. Both groups received intravenous infusions of 1.0 grams of tranexamic acid pre- and post-surgery, with an additional 1.0 grams locally injected following joint cavity closure during the procedure. Li Jun et al^[17]^ reported that combining intravenous and local administration of tranexamic acid effectively reduces perioperative blood loss in THA without elevating the risk of postoperative deep vein thrombosis or pulmonary embolism. Excessive intraoperative soft tissue debridement, such as in cases of thickened hip joint synovium or scar hyperplasia, should be minimized to prevent increased blood loss while maintaining anatomical clarity. Studies^[18]^ have shown that the postoperative decrease in hemoglobin levels following THA does not correspond to the amount of blood lost during the surgery, indicating a significant amount of concealed blood loss post-operation. Sehat et al^[19]^ reported a hidden blood loss of 471 mL after THA, constituting approximately 26% of total blood loss. Research on hidden blood loss remains inconclusive, yet it is linked to various factors including anesthesia type, age, gender, BMI, underlying diseases, fracture type, surgical approach, prosthesis type, and anticoagulant use.^[20]^ During the perioperative period, regular monitoring of hemoglobin levels is essential, and proactive measures should be implemented early to mitigate risk factors, thereby reducing the likelihood of occult blood loss and enhancing patient prognosis.

### 4.1. Limitations

This study has several limitations. First, it was retrospective and non-randomized, and the choice of surgical approach was made in routine clinical practice rather than assigned by protocol, which may have introduced selection bias. Second, although we added adjusted longitudinal and multivariable analyses, residual confounding cannot be excluded because not all potentially relevant perioperative, indication-related, and surgeon- or center-level variables were available for modeling. Third, each participating center contributed one senior surgeon, which reduced within-center variability but also means that center effects and surgeon effects could not be fully disentangled. Finally, the study mainly assessed early and short-term outcomes, and longer follow-up is needed to determine the medium- and long-term value of the Mis-PLA approach in more complex arthroplasty scenarios.

## 5. Conclusions

In this retrospective multicenter study, preservation of the piriformis muscle during posterolateral THA was associated with a lower incidence of early postoperative dislocation and better early functional recovery. There was no significant difference in 12-month Harris Hip Scores between groups. These findings support the feasibility of the piriformis-sparing posterolateral approach, although the results should be interpreted cautiously given the observational design and the potential for residual confounding.

## Author contributions

**Conceptualization:** Kaiyuan Zheng, Haiqi Ding, Jiuzao Lin, Jiagu Huang.

**Data curation:** Changzhi Huang, Bei Li, Naijian Dong.

**Writing – original draft:** Kaiyuan Zheng, Haiqi Ding, Xiaoyong Wang.

**Writing – review & editing:** Jie Xu, Jiuzao Lin, Jiagu Huang.
